# Supplementary material for: Are carbon emissions trading and green financial instruments synergistic? -Comprehensive quantitative research based on content analysis
Source: PLoS One. 2024 Mar 7;19(3):e0298601. doi: 10.1371/journal.pone.0298601 (PMC10919601; doi:10.1371/journal.pone.0298601)
Supplement: S1 Data — (ZIP) [file pone.0298601.s001.zip › Data/地级市Z_F工作报告.zip!/─┌├╔╣┼/╢⌡╢√╢α╦╣╩╨/└┤╫╘╣1⁄2╓┌║┼í╛┬φ┐╦╩2╛▌═°í┐_2015.docx]

各位代表：

　　现在，我代表市人民政府向大会报告工作，请予审议，并请市政协委员和列席会议的同志们提出意见。

　　一、2014年工作回顾

　　过去的一年，是我市发展历程中极不平凡的一年。面对经济持续下行压力，市人民政府在市委的坚强领导下，在市人大常委会和市政协的监督支持下，团结带领全市各族人民，认真贯彻落实中央、自治区和市委的各项决策部署，统筹推进稳增长、调结构、促改革、惠民生、防风险各项工作，较好地完成了市三届二次人代会确定的目标任务，经济社会发展取得新成效。

　　（一）经济实现平稳增长。全市地区生产总值突破4000亿元，达到4162.2亿元，增长8%；规模以上工业增加值突破2000亿元，达到2058.8亿元，增长11.1%；固定资产投资突破3000亿元，达到3422亿元，增长15.8%；公共财政预算收入完成430.1亿元，下降2.3%；社会消费品零售总额完成610亿元，增长10.1%。全力推进项目建设促增长，蒙西至天津南电力通道、伊泰煤制油等21个项目获得国家和自治区核准或出具“路条”，总投资1458亿元；开复工亿元以上重大项目175项，中煤大化肥、汇能煤制气、蒙西粉煤灰提取氧化铝等25个项目建成投产。全力扶企业、稳工业，出台了稳定企业生产经营、促进非公经济发展等政策措施，减轻企业税费负担23亿元，落实电价、职工培训等补贴8.1亿元；电力多边交易企业达到20户，交易电量89.2亿千瓦时，降低企业生产成本2.2亿元；积极帮助企业拓展市场，增加煤炭供需协议量4053万吨。规模以上工业企业实现主营业务收入4277亿元、利润608亿元、税金353亿元，均居全区第一位。工业用电量、公路和铁路货运量分别增长12.9%、27.5%和12%。

　　（二）转型升级成效明显。工业经济转型步伐加快，资源型产业延伸升级、非资源型产业规模化发展都取得新进展，新增煤化工产能190万吨；非煤产业完成投资1989亿元，占工业总投资的80%，实现增加值869亿元，占规模以上工业增加值的42%，同比提高6.2个百分点；煤炭产业占工业的比重降到60%以下。农牧业稳定发展,粮食产量30亿斤，牧业年度牲畜存栏1227万头只，新建农业示范基地16万亩，培育养殖大户250户、市级以上龙头企业31家。一产实现增加值99.6亿元,增长4.1%。现代服务业加快发展，文化旅游、休闲度假、健康养生、电子商务等成为新的消费热点。旅游业实现收入190亿元，增长29.3%；金融机构存贷款余额分别达到2494亿元和2589亿元，增长7.3%和9.3%。三产实现增加值1599.6亿元，增长5.1%。

　　（三）改革创新力度加大。行政审批、工商登记、财税金融、资源配置、农村牧区等领域改革扎实推进。在全区率先公布市本级行政权力清单和负面清单，清理、取消和下放行政审批事项202项。市场主体活力进一步增强，新登记各类企业和工商户2.8万户，民间投资增长17.3%，营业收入超百亿元非公有制企业达到16户。土地确权登记、草牧场划定试点工作进展顺利，流转土地、草牧场71.3万亩。我市被确定为国家资源型经济创新发展综合改革试点、全国首批生态文明先行示范区，被自治区确定为主要污染物排污权有偿使用和交易试点。科技创新力度加大，组织实施重大技术成果转化项目27项，新增高新技术企业8家、科研机构6家，通过国家可持续发展试验区复审验收。

　　（四）统筹发展步伐加快。城市功能进一步完善，完成市政基础设施和园林绿化投资50.6亿元，新增绿地993万平方米，新建改造市政道路265万平方米，铺设燃气、供水等管网275公里，中心城区公交实现一体化运营，市体育中心、阿布亥沟治理等工程加快推进，建成国家园林城市。农村牧区“十个全覆盖”工程扎实推进，完成投资20.5亿元，其中改造危旧房10350户，硬化街巷道527公里，11.5万户群众实现广播电视户户通，解决了5.5万人饮水安全问题。交通、水利、电力等基础设施建设力度加大，完成投资189亿元，新增铁路运营里程126公里、高等级公路73公里、通村油路467公里，架设110千伏以上输变电线路218公里。鄂前旗城乡统筹、杭锦旗扶贫开发、达旗县域经济发展示范区建设稳步推进，区域协调发展步伐加快。

　　（五）生态环境持续改善。大气污染防治成效明显，空气质量优良率达到81.8%。严格水资源管理，关闭工业自备水源井162眼。启动“数字环保”工程，实现对重点行业、重点企业实时监控。强力推进环境综合整治，对突出环境隐患问题进行限期整改，取缔关闭、停产整顿违法企业198家。淘汰电石、焦化、水泥等落后产能103万吨，新建污水处理厂12座，削减二氧化硫5.3万吨、氮氧化物6.4万吨、化学需氧量1500吨、氨氮300吨，单位GDP能耗下降3.2%，完成年度节能减排任务。加强生态保护与建设，强化重点区域绿化，完成林业生态建设150万亩、草原建设743万亩、水土流失治理180.5万亩，全市森林覆盖率达到25.8%。

　　（六）民生保障更加有力。完成民生和社会事业投入442亿元，占公共财政预算支出的81.6%。城乡常住居民人均可支配收入分别增长8.5%和11%，达到34983元和13439元。十个方面惠民实事有效落实。建成大学生、农牧民创业园，“零就业”家庭至少1人稳定就业，新增城镇就业3.4万人，城镇登记失业率控制在2.61%。企业退休人员养老金人均每月提高182元，城乡居民低保标准分别提高15元和12元、医保筹资水平分别达到597元和610元。为21858名残疾人发放补贴3228万元。新建改扩建中小学幼儿园57所、标准化卫生室94个，成为国家公立医院改革试点。国家公共文化服务体系示范区建设扎实推进，新建续建嘎查村文化活动室108个，为120个文化室配备设施设备。《警察日记》和《小牛向前冲》两部作品荣获全国“五个一”工程奖。实施“单独两孩”政策，人口出生率控制在10.35‰。精准扶贫扎实推进，2.6万贫困人口稳定脱贫。回购、新建保障性住房12934套。发放农牧户冬季用煤补贴2.34亿元、低保家庭和蒙古族大学生入学补助资金6000余万元，39万户农牧民、9139名大学生受益。

　　（七）社会大局保持稳定。积极防范化解金融风险,争取国家银监会和自治区政府出台了银行业支持鄂尔多斯转型发展政策，全年新增社会融资760.8亿元，协调金融机构对435亿元贷款予以续贷和展期，有效降低企业金融风险。严厉打击非法集资活动，部分民间借贷案件得到化解。通过回购、促销等措施，消化存量商品房294万平方米。出台政府性债务管理办法，完成清理甄别工作，净化解政府性债务165.9亿元。信访、社会治安、安全生产形势稳定，信访总量、刑事案件、生产经营性事故分别下降24.5%、9.5%和44.9%。

　　（八）政府自身建设得到加强。扎实开展党的群众路线教育实践活动，认真落实作风建设各项规定，严格执行“约法三章”，坚决整治“四风”问题，政府系统作风明显转变。市本级清理规范性文件273件、临时议事机构500个、表彰奖励项目25项，“三公经费”压减53%。全市清理超标超配公车3475辆、超标办公用房18.9万平方米。自觉接受人大、政协和社会各界监督，办结人大代表建议和政协委员提案279件。通过开展电视问政、开通政务微博，推动重大民生问题解决落实。审计、监察和政务公开力度加大，出台了财政支出审批、国有资产处置、机关差旅费支出、公共资源交易等一系列管理制度，市直95个预算部门和大部分旗区、园区公开“三公经费”预决算，廉政建设和反腐败工作取得新成效。

　　此外，精神文明和民主法制建设得到加强，顺利通过第四届全国文明城市复查测评。第十届全国少数民族传统体育运动会筹备工作有序推进。国防动员、人民防空、民族宗教、新闻广电、外事侨务、史志档案、气象地震、妇女儿童、老龄事业、对口支援兴安盟等各项工作均取得新成效。

　　各位代表，过去的一年，困难比预料的多，结果比预想的好，取得这样的成绩，确实来之不易。这是自治区党委、政府和市委正确领导的结果，是市人大、政协大力支持的结果，是全市人民团结拼搏、积极进取的结果。过去一年的工作实践，使我们进一步加深了对推进转型发展重要性和紧迫性的认识，更加坚定了把鄂尔多斯建成祖国北疆亮丽风景线上璀璨明珠的信心和决心。在此，我代表市人民政府，向全市各族干部群众和所有关心、支持、参与鄂尔多斯建设发展的社会各界人士，表示衷心的感谢并致以崇高的敬意！

　　同时，我们也清醒地认识到，当前我市发展中也面临着不少困难和问题。主要是：受市场需求不振影响，部分工业产品价格下降，投资增长难度加大，企业生产经营困难，财政收支矛盾比较突出，经济下行压力仍然较大；产业链条短，产业结构单一，调结构、促转型的任务十分繁重；农村牧区基础设施和公共服务水平还不高，统筹城乡发展的任务仍然艰巨；金融、民间借贷、房地产、政府性债务等领域的风险需进一步化解；政府职能转变和作风改进还不够到位，依法行政水平和办事效率仍需提高，等等。这些问题，我们将采取有力措施，认真加以解决。

　　二、2015年主要工作任务

　　今年是全面深化改革的关键之年，是全面推进依法治市的开局之年，是新常态下推动转型发展的攻坚之年，也是全面完成“十二五”规划的收官之年，做好今年各项工作，意义十分重大。当前，我国经济发展进入新常态，呈现速度换挡、结构优化、动力转换的新特征，我们面临的需求、供给、产业组织、市场竞争等外部环境发生重大变化，稳增长、调结构任务十分艰巨。新常态有新挑战，更蕴含新机遇。国家调整经济结构、治理大气污染，为我们推进产业转型特别是清洁能源产业发展提供了广阔市场空间；国家实施积极财政政策，扩大中西部地区基础设施等领域投资规模，为我们突破要素制约、加强基础建设提供了难得的政策机遇；国家和自治区持续简政放权、深化改革、推进创新，特别是支持我市资源型经济创新发展和生态文明先行示范区建设，为我们释放改革红利、激发创新潜力、增强发展动力创造了有利条件。自治区从项目、资金、资源等各方面都给予我市大力支持。我市经过多年发展，形成了坚实的产业基础和较为完善的基础设施体系，一批调结构、促转型的大项目正在建设，发展潜力巨大、前景广阔。总的看，新常态下我市仍处于大有作为的战略机遇期。我们要充分认识新常态，主动适应新常态，积极引领新常态，坚定信心，振奋精神，因势利导，主动作为，努力开创新常态下转型发展新局面。

　　今年政府工作的总体思路是：深入贯彻中央、自治区各项决策部署，全面落实市委三届五次、六次全委会议精神，坚持稳中求进工作总基调，坚持以提高经济发展质量和效益为中心，主动适应经济发展新常态，强化持续发展、转型发展、协调发展、和谐发展理念，着力稳增长，着力调结构，着力促改革，着力惠民生，着力防风险，扎实推进法治政府建设，不断增强鄂尔多斯发展动力、活力和竞争力，推动经济社会平稳健康发展。

　　经济社会发展的主要预期目标是：地区生产总值增长8%左右，固定资产投资增长13%以上，规模以上工业增加值增长11%左右，公共财政预算收入增长3%左右，社会消费品零售总额增长11%左右，城乡常住居民人均可支配收入分别增长9%和10%以上。居民消费价格指数涨幅控制在3%以内，城镇登记失业率控制在3.5%以内。完成自治区下达的节能减排目标任务。

　　这些预期目标的确定，综合考虑了经济发展新常态下我市发展的新趋势新变化,与“十二五”规划和全面建成小康社会目标相衔接，符合市场预期，符合经济发展规律，体现了中央、自治区稳中求进的工作总要求，兼顾了稳增长与调结构的需要，是实事求是、积极稳妥的。实现这些目标，也需要付出艰辛努力。我们将凝聚各方面力量，扎实推进各项工作，努力实现这些目标任务。

　　今年，市政府将重点做好七个方面工作：

　　（一）牢牢把握稳中求进工作总基调，保持经济稳定增长。

　　毫不放松抓项目扩投资。新常态下，项目带动、投资拉动仍将是我市经济增长的主要动力。今年实施亿元以上重点项目192项，其中新建78项。要全力突破项目审批和用水、用地、环保、能耗等要素制约，加大闲置指标清理力度，保障重点项目建设，力促项目早获批、早开工、早投产、早达效。强化以商招商、定向招商，建立专业化、链条化、集群化招商机制，引进承接一批战略性新兴产业和非资源型产业项目，力争引进国内区外资金700亿元以上。建立市旗两级项目储备联动机制，围绕“五大基地”建设，策划、储备一批产业链延伸配套、现代服务业、现代农牧业项目，保持项目建设后劲。抓住国家支持基础设施建设、能源蠢嘞钅肯蛑形鞑康厍兔褡宓厍畔炔季值幕觯≈氐悴怠⒒∩枋⑸缁崾乱档攘煊蛳钅亢屯蹲省＜哟蟛普龀至Χ龋镒?span lang="EN-US">30亿元设立转型发展基金，引导产业转型升级。通过特许经营、投资补助等措施，撬动更多社会资本参与投资。优化投资结构，扩大中高端产业、终端化和高质化产品项目投资，努力培育新的经济增长点。全年500万元以上项目完成固定资产投资2800亿元以上。

　　全力以赴稳工业扶企业。加强工业经济运行调度，落实国家和自治区促进工业转型、稳定煤炭运行等扶持政策，保障工业经济稳定发展，500万元以上工业项目完成投资2000亿元以上，确保30个重点项目建成投产。利用我市区位优势和煤炭品质，建立标准化质量体系，打造“鄂尔多斯煤”国优品牌。抓住国家煤炭限产保价、鼓励出口、提高进口煤准入标准等机遇，推动地方煤炭企业与五大电力公司等用煤企业深度合作、战略重组，实施煤电联合发展，全力保价、保量、保质，扩大我市煤炭市场份额和话语权。新增煤炭洗选量3000万吨。深入实施“服务企业工程”，落实国家、自治区和我市煤电油运等优惠政策，积极争取国家调整硅铁出口关税、煤制油产业差别化消费税等税收优惠和汽车产业扶持政策，坚决清理不合理收费，降低企业生产成本，为企业“松绑减负”。落实稳定岗位补贴政策，对不裁员、少裁员的困难企业给予社会保险、转岗培训、技能培训补贴。加强职业技能培训，帮助企业解决招工难、用工难问题。实施大型骨干企业培育计划，更加注重扶持中小微企业配套发展，鼓励引导企业进行技术升级改造和整合重组，激发活力、稳定生产、提升竞争力。新培育销售收入超50亿元大型企业5户以上，新注册中小微企业5000户以上。

　　千方百计促增收保运转。落实好煤炭资源税从价计征、“营改增”等政策，挖掘资源清理、煤炭运销、油气输送、企业属地注册、股权转让等领域税收增长潜力，积极争取上级财政支持政策和各类专项资金。优化税收结构，提高税收质量，增加地方可用财力。加强企业服务，促进项目建设，做大税基规模，努力培植新的财源增长点。坚持有保有压、有扶有控，优化财政支出结构，财政资金重点保运转、保民生、保化债、保重点续建项目。牢固树立长期过“紧日子”思想，坚持勤俭办一切事业，严格控制一般性支出和政府投资类项目规模，今年“三公”经费在上年基础上再压减5%以上，除民生项目外仍不开工建设新的政府投资类项目。健全财政支出绩效评价机制，强化财政资金使用监管，把有限的资金用在刀刃上，提高资金使用效益。

　　（二）大力调整优化经济结构，提高经济增长质量和效益。

　　推动资源型产业延伸升级。立足资源调结构、促转型，坚定不移发展壮大清洁能源和现代煤化工产业。加快煤电基地建设，力争蒙西至天津南特高压输电通道上半年开工建设，上海庙至山东特高压输电通道及配套电源点项目早核准早开工，确保蒙泰、君正等热电厂项目建成投产，新增电力装机340万千瓦以上。加快推进神华煤制油第二、三条生产线、伊泰精细化学品等项目建设，争取新蒙、北控、中海油、河北建投、华星煤制气和伊泰200万吨煤制油等项目核准并开工建设。要在做精做优煤化工产业上下功夫，控制初级加工产品生产规模，推动煤化工产业延伸链条，争取中电投80万吨烯烃项目年内核准，加快推进中天合创甲醇转烯烃等项目建设，确保久泰60万吨、中煤蒙大60万吨烯烃、亿利一期30万吨乙二醇等项目建成投产，新增烯烃产能120万吨以上。

　　促进非煤产业集群配套发展。紧盯市场需求变化，大力发展装备制造、电子信息等产业，着力在扩大规模、聚集产业、完善配套、引进高新技术上下功夫、见实效。保障奇瑞、华泰等汽车企业稳定生产，重点推动产业配套，新引进零部件配套生产企业10家以上，力争奇瑞35万台发动机、15万台变速器项目达产。抓好江苏工业园区建设，引进承接一批装备制造等产业项目。围绕主导产业，配套发展煤机、化机和电力装备等制造业。加快发展电子产品制造、云计算和大数据产业，重点推进罗克佳华物联网和毅航、中兴云计算等项目建设，培育电子信息产业集群。推进高铝粉煤灰资源综合利用，保障蒙西氧化铝等项目达产达效，加快神华、大唐、中铝、鄂绒等项目建设，形成120万吨氧化铝生产规模。出台振兴羊绒产业政策，依托鄂尔多斯羊绒产业园区，推进产业整合重组和优化升级，全面振兴羊绒产业，再创鄂尔多斯羊绒产业发展新辉煌。稳步发展光伏产业，推动建材、陶瓷、PVC等产业高端化、品牌化、系列化发展。全市非煤产业增加值突破1000亿元，占规模以上工业增加值比重提高到45%以上。

　　提升现代服务业发展水平。把服务业作为调结构、促转型的重要突破口，重点发展文化旅游、现代物流、健康养老等产业，大力发展电子商务新业态，促进服务业发展提速、比重提高、结构优化。推进文化旅游资源深度开发，申报国家旅游综合改革试点城市，集中打造成陵、响沙湾、康巴什、七星湖等旅游景区，大力开发城市、工业和农家乐、牧家游等旅游产品，推动蒙古源流、天风动漫、蒙元文化影视城等文化项目建设，培育动漫、影视、微电影等文化新业态，完成文化、旅游投入20亿元以上，游客数量同比增长10%以上。落实物流业发展规划和扶持政策，依托城镇和园区，加快培育一批配套物流企业，今年新建2至3个大型物流市场，启动建设奥特莱斯航空城项目。大力发展健康养老产业，鼓励社会力量兴办医疗保健、康复护理、休闲度假等项目，重点打造东胜健康产业集中发展示范区。适应新常态下个性化、多样化消费和大众创业需求，抓住建设国家信息消费试点城市机遇，引进建设阿里巴巴、京东等大型电商平台，筹建“淘宝地方特色馆”，启动建设鄂尔多斯电子商务网和第三方支付平台，鼓励更多企业和个人开办网店，扩大“鄂尔多斯产品”销售，促进整体经济与新型互联网经济融合。全年服务业增加值增长6.5%左右。

　　加快转变农牧业发展方式。坚持走绿色高端、以质取胜、品牌增收的路子，调整优化种养结构、产品结构、区域结构，集中打造沿黄河、无定河现代农牧业经济带和西部现代草原畜牧业示范区，建设绿色农畜产品生产加工输出基地。继续推进土地流转，探索土地股份合作、租赁经营等新模式，扩大规模经营面积。设立农牧业转型发展基金，捆绑使用涉农涉牧资金，扶持肉牛、肉羊、生猪、瓜果蔬菜、林沙等优势产业做大做强，打造鄂尔多斯农畜产品绿色品牌。新建公司化养殖园区14个，种植瓜果蔬菜30万亩，新培育现代家庭牧场200户。实施耕地质量保护和提升行动，推广节肥、节药、节水和清洁生产技术，促进农牧业绿色无公害、可持续发展，新建绿色食品原料基地20万亩。加强“菜篮子”工程建设，强化技术指导推广，提高设施农业发展水平。积极培育龙头企业、专业合作社、家庭牧场等新型经营主体，力争在每个优势产业领域培育1至2家大型龙头企业，构建种养加销全产业链。今年重点推进五家尧食用菌、飞鹤乳制品、正大食品生产加工等项目建设，新增市级以上龙头企业26家。完善龙头企业与农牧户利益联结机制，通过产业化促进农牧民增收。

　　（三）全面推进改革创新，增强持续发展动力、活力和竞争力。

　　深化重点领域改革。以建设资源型经济创新发展综合改革试点和生态文明先行示范区为抓手，从制约地区发展的突出问题和关键环节入手进行攻坚突破，重视改革方案设计，加快改革进度，增强改革效果。继续简政放权，清理、取消和下放行政审批事项，今年要再下放一批行政审批项目。放宽市场准入，推行“三单”管理模式，扩大“三证合一”登记制度试点范围。深化财税体制改革，健全全口径预算体系，推行财政预算绩效管理制度。建立政府购买公共服务目录，扩大购买公共服务覆盖面。启动不动产统一登记工作。加快资源资本化进程，深化市直国有企业改革。支持非公有制、混合所有制经济发展，推广政府和社会资本合作模式。启动棋盘井电力微网运行试点，开展水权、碳排放权、排污权交易，完成煤炭资源配置清理工作任务。推动金融综合改革，争取获批1家民营银行。深化文化事业单位改革，加快建立法人治理结构。落实最严格的水资源管理制度，推动灌区水权细化试点改革和农业、居民用水阶梯水价改革，建设节水型社会。探索编制自然资源资产负债表，建立资源环境承载能力监测预警制度。推进农村牧区产权制度改革，争取把更多的静态资产变为流动资本。完成农村牧区土地确权登记颁证、草原确权承包试点及基本草原划定任务。抓好达旗“扩权强县”试点和县域经济发展示范区建设，巩固提升鄂前旗城乡统筹示范区建设水平。把扩大开放作为深化改革的重要内容，积极争取建设鄂尔多斯综合保税区，大力发展外向型经济，培育新的经济增长点。

　　加强创新能力建设。适应经济发展新常态，推动经济增长由要素驱动向创新驱动转变。深入实施“草原英才”工程和“十万大学生创业就业圆梦行动”，培养和引进一批创新型人才。加强与高等院校和科研院所合作，推进市高新技术产业园区、清洁能源国际创新园建设。强化政策扶持与引导，加快培育一批掌握核心技术、引领行业发展的创新型大企业，发展一批具有自主知识产权的中小型企业。组织实施重大科技专项，扶持企业与科研机构联合开展技术攻关，解决煤化工产业高耗煤、高耗水、高碳排放技术问题，争取在煤化工产业链延伸、粉煤灰提取氧化铝、装备制造、电子信息等领域突破一批核心关键技术，力争把我市建成清洁能源和煤化工技术示范基地。

　　（四）持续加大民生改善力度，织密织牢民生保障网。

　　切实保障特殊困难群体的基本生活。多做“雪中送炭”的工作，今年整合惠民政策和资金，重点实施六个方面民生工程：一是扎实推进创业就业，新增城镇就业3.2万人，其中安置鄂尔多斯籍高校毕业生1万人；发放小额贷款2.5亿元，扶持5000人创业。二是全面推进“十个全覆盖”工程，投资41.9亿元，完成三年工程量的80%以上，改造农村牧区危旧房3万户，其中沿黄渗漏区4000户，硬化街巷道1179公里，改造农网线路9290公里，解决9.4万人饮水安全问题，为6.4万户群众安装广播电视户户通设备，新建改扩建嘎查村标准化卫生室85个、文化室131个、便民连锁超市100个。三是深入推进扶贫攻坚工程，加大精准扶贫力度，重点抓好杭锦旗扶贫开发示范区建设，全市实现2.4万贫困人口稳定脱贫。继续实施农村牧区低收入家庭免费发放取暖煤政策。四是加强保障性安居工程建设，以回购为主新增各类保障性住房22478套，其中棚户区改造19206户。五是进一步提高社会保障标准，将企业退休人员基本养老金提高10%，城乡居民最低生活保障标准每人每月提高15元和12元、医疗保险筹资标准提高60元和46元、养老金待遇标准各提高15元。六是新建、续建、改扩建中小学校21所、幼儿园37所，在落实自治区低保家庭大学生入学资助政策的基础上，对鄂尔多斯籍家庭经济困难大学生考取专科、本科、研究生的分别给予3000元、4000元和5000元资助金，保障困难家庭学生上得起学、上得好学。

　　提高社会事业发展水平。统筹推进贫困地区义务教育学校标准化建设，加快改善薄弱学校办学条件。深化教育教学改革，利用信息化手段推动基础教育均衡发展，提高办学质量和水平，年内准旗、伊旗、鄂前旗通过国家县域义务教育均衡发展达标评估验收。实施“立德强能”工程，提高学生综合素质。深化职业教育改革，推进产教融合、校企合作，完善现代职业教育体系。整合高等教育资源，组建鄂尔多斯应用技术学院。大力推进公立医院改革，完善重大疾病医疗保险和救助制度，优化城乡医疗资源配置，通过免费培训和脱产进修，提高农村牧区医务人员的业务水平，让农牧民群众享受到高质量的医疗服务。巩固国家公共文化服务体系示范区创建成果,实施文化信息资源共享、数字图书馆、公共电子阅览室等数字文化工程。大力发展足球事业，启动校园、社区、企业足球联赛。统筹利用现有文体场馆，开展群众性文体活动。承办好第十五届全国青少年机器人大赛。保障妇女儿童权益，做好人口计生、外事侨务、新闻广电、老龄事业等各项工作。

　　（五）着力推动城乡基础设施建设，提高城乡统筹发展水平。

　　推进品质城市建设。严控城市建设规模，进一步完善功能、提升品位、加强管理，推动城市建设从规模扩张向质量提升转变。强化规划统筹，推进城镇“多规合一”建设管理。加快推进中心城区一体化发展，统筹产业布局、社会事业发展和基础设施建设，今年完成市政建设投资50亿元。加大城市绿化力度，强化养护管理，巩固园林城市创建成果，新增城市绿化面积500万平方米。大力发展总部经济和楼宇经济，加快聚集人气、商气。出台户籍制度改革配套政策，吸引更多人口到中心城区创业发展、就业生活。推进智慧城市建设，今年实现中心城区无线网络全覆盖。加强物业管理，提升城市精细化管理水平。加快小城镇建设，增强综合承载能力。建立城镇地下管线综合规划管理机制，完成地下管线普查工作。统筹城镇、园区规划建设，推动产城融合互动发展。

　　改善农村牧区发展环境。编制全市农村牧区村庄布局规划，更好地指导和推动村庄建设发展。结合农村牧区“六位一体”工程实施，同步推进村庄绿化、村集体经济和基层组织“三项建设”。进一步改善农村牧区基础设施条件，实施一批中小型农田水利工程，新建改建通村油路700公里。推进新农村新牧区建设，实施“城乡洁净”工程，完善垃圾、污水处理等设施，整治村容村貌，建设美丽乡村。稳步推进农牧民转移，逐步改善禁止开发区内未转移农牧民的生产生活条件。

　　提升基础设施承载能力。开工建设棋盘井至乌海、纳日松至龙口、敖勒召其至东道梁、嘎鲁图至乌兰镇等高等级公路，启动沿黄一级、包茂高速改扩建工程，确保荣乌高速十七沟至大饭铺、东胜至机场高速公路建成通车，新增高等级公路122公里。开工建设蒙西至华中运煤通道我市境内工程，加快呼准鄂等铁路建设，确保沿河铁路、东乌与包西连络线、鄂尔多斯火车站建成投运，推动包西高速客运专线建设项目纳入国家“十三五”规划，年内新增铁路运营里程300公里。开工建设杭锦旗通用机场。根据产业发展和项目建设进展情况，统筹推进各园区内外基础设施建设，特别是加快独贵塔拉、三垧梁、图克等工业园区供水工程建设，完成水权转换二期工程，提升园区综合承载能力。

　　（六）进一步加强生态文明建设，筑牢绿色安全发展屏障。

　　坚决整治环境污染。认真执行新的环保法，落实最严格的监管责任，实施环境保护综合整治三年行动计划，突出抓好大气、水、土壤、重金属污染防治和主要污染物减排，最大限度减少环境负债。严把环保准入关，制定产业发展目录，严格控制高污染、高排放、高耗水项目建设，对环保违规项目进行全面清理。突出抓好矿区环境综合整治，加强园区和城镇污水、垃圾、高盐水处理厂等环保设施建设，完成神华煤制油项目用水置换、毛莲圪卜污水圈和骆驼山矿区环境综合整治任务。继续淘汰落后产能，大力发展循环经济，提高煤矸石、煤泥、粉煤灰和矿井疏干水、城市中水综合利用水平。强化环保能力建设，对环境污染问题出重拳、施重手，进行严肃整治。持续推进环保模范城市创建，保护好“天蓝、地绿、水清、气净”的生态环境。

　　加强生态保护与建设。严格执行农村牧区“三区”发展规划和禁牧、休牧、划区轮牧政策，设立草原保护红线，严禁开荒扩灌，巩固生态建设成果。推进城区、矿区、交通沿线、旅游景区、村庄周边等重点区域绿化，促进生态脆弱区自然修复，实施京津风沙源治理、十大孔兑综合治理等重点生态建设工程，启动农牧民户植百棵树行动，完成林业生态建设130万亩、草原建设960万亩、水土流失治理180万亩，建成国家森林城市，最大限度增加生态资产。

　　（七）不断加强和创新社会管理，保持社会和谐稳定。

　　严密防范化解各类风险。全面落实自治区银行业支持鄂尔多斯转型发展措施和财政金融互助政策，争取更多的企业和项目得到金融支持。鼓励企业通过上市、股权投资、引进保险资金和发行中期票据、短期融资券、中小企业集合债券等形式多元融资，有效舒缓债务链条，新增融资规模500亿元以上。积极参与自治区资产管理公司组建，盘活存量资产。推动龙头企业、优质企业对不具备竞争优势、资金链紧张的出险企业进行兼并重组，降低企业金融风险。开展打击恶意逃废金融债务专项行动，确保金融形势稳定。严格落实政府性债务管理办法，通过预算安排、股权合作、资产拍卖等措施，加快化解存量政府性债务，确保债务余额在上年基础上再下降20%。坚持用法治思维和方式解决民间借贷问题，加大非法集资案件查处力度，严厉打击隐匿资产、高价抵顶、恶意逃债、有钱不还等赖账行为，依法保障群众合法权益。促进房地产业稳定发展，通过团购促销、回购拉动、发展旅游和养老地产等措施，消化存量商品房150万平方米以上。

　　加强社会治安综合治理。推进国家社会管理创新试点城市建设，健全矛盾纠纷排查化解机制，推行网上信访，提高信访信息化、法治化水平，依法处置土地征用、房屋拆迁、环境污染等领域矛盾问题。严格落实安全生产“党政同责、一岗双责、齐抓共管”和企业主体、政府监管责任，加强安全设施建设和技能培训，强化矿山、冶金、危化企业、道路交通、油气管道、消防等重点领域以及人员密集场所安全监管，实行化工行业专家巡查和分级分类监管，从源头上防止安全事故发生。强化食品药品安全监管，强化监测检验能力建设，完善食品质量安全追溯体系，为每个苏木乡镇食品药品监督所配备一台监督执法车辆，保障人民群众“舌尖上的安全”。深化平安鄂尔多斯建设，加强社会治安综合治理，严厉打击各类违法犯罪活动，加大禁毒、反恐工作力度，提升治安防控立体化、网络化、信息化水平。推进社会诚信体系建设，实行诚信“红黑名单”发布及奖惩制度，塑造诚信鄂尔多斯形象。加强应急队伍建设和救援演练，提高突发事件处置和自然灾害应对能力。强化国防宣传教育，巩固双拥模范城市创建成果。全面贯彻中央、自治区和我市民族工作会议精神，落实支持少数民族和民族聚居地区发展的政策措施，推动我市民族团结进步事业蓬勃发展，维护各族人民团结奋斗、安居乐业的大好局面。

　　进一步提升城市文明水平。大力弘扬“开放包容、诚信友善、不屈不挠、拼搏创新、艰苦奋斗、不断进取”的鄂尔多斯优秀精神品质，抓好“爱我鄂尔多斯”主题教育实践活动。积极践行社会主义核心价值观，进一步提升市民素质。扎实开展全国文明城市创建工作，巩固和提升文明城市建设水平。

　　各位代表，今年8月份第十届全国少数民族传统体育运动会将在我市举办，这既是国家和自治区交给我市的一项光荣使命，也是提升我市美誉度和影响力的一次重大机遇。我们要精心组织、认真筹备，扎实做好场馆建设、赛事安排、环境整治、宣传动员等各项工作，确保承办一届安全、团结、节俭、热烈的民族体育盛会，充分展示鄂尔多斯良好形象，激发全市人民干事创业的激情和热情，努力让办会成果惠及人民群众。另外，我们将着眼于经济发展新常态，科学编制“十三五”规划，为我市持续健康发展提供有力支撑。

　　三、切实加强政府自身建设

　　适应新常态，谋求新发展，完成新任务，对政府自身建设提出了新的更高要求，需要各级政府顺势而为、主动作为、奋发有为。

　　严格依法行政。全面贯彻党的十八届四中全会精神，建设法治政府，推进依法治市。坚决执行人大及其常委会的决议、决定，自觉接受人大法律监督和政协民主监督，支持各民主党派开展工作。坚持法定职责必须为、法无授权不可为，依法履行政府职能。完善公众参与、专家论证、风险评估、合法性审查、集体讨论决定等机制，提高行政决策水平。整合执法资源，减少执法层级，推行综合执法，解决多头执法、多层执法等问题。强化执法培训与管理，建立执法全过程记录制度，规范行政许可、行政处罚、行政强制、行政检查等程序。加大食品药品、安全生产、环境保护等重点领域执法力度，切实保障群众切身利益。推进“六五”普法，加强法制宣传教育，提高全民法治意识，改善地区法治环境。

　　持续改进作风。巩固和扩大党的群众路线教育实践活动成果，扎实开展“三严三实”主题实践活动。严格执行中央“八项规定”、国务院“约法三章”和自治区、市委各项配套规定，持之以恒整治“四风”，驰而不息转变作风。继续精简会议和文件，严控各类会展、论坛、节庆和检查评比活动。完成公务用车制度改革任务，统筹利用腾退办公用房。落实从严治政责任，严格公务员队伍管理，严肃查处对待群众简单粗暴、作风蛮横、以权谋私等行为。深入推进电子政务建设，全面推行网上审批、并联审批和代办服务，市旗两级行政许可事项全部进驻政务服务大厅集中办理。扩大行政权力运行、公共资源交易等领域信息公开范围，规范细化公开内容，年内市旗两级财政预决算、部门预决算、“三公经费”预决算全部公开。完善政府新闻发布制度，利用微博、微信等新媒体及时回应社会关切，提高政府工作透明度。

　　提高执行能力。全面落实市委加强执行力推动抓落实的规定，严明执行责任，加强履责监管，保障重大决策、重大项目、重点工作落到实处。深化机关效能建设，发扬苦干实干精神，坚决纠正不作为、慢作为、乱作为，坚决整治“混日子”、“守摊子”、“为官不为”等懒政惰政怠政现象，营造想干事、会干事、干成事氛围。直面问题、精准发力，着力研究解决经济发展和社会治理等领域矛盾问题。大力开展行风政风评议，进一步改进机关作风。严格行政问责，坚决查处执行不力、失职渎职、弄虚作假等行为，保障政令畅通，确保各项工作落到实处。

　　加强廉政建设。严格落实党风廉政建设各项规定，强化政府内部权力监督制约，在权力集中部门和重点岗位探索推行分事行权、分岗设权、分级授权。依法规范招投标活动，严肃查处平台外交易和围标串标等行为。强化政府投资项目监管，严控工程建设规模标准，坚决避免重复建设和超预算、超标准建设行为，不准盲目铺摊子、上项目，杜绝形象工程、面子工程。加强审计监察，严肃查处吃拿卡要、暗箱操作、权力寻租、贪污腐败等违法违纪行为，努力做到干部清正、政府清廉、政治清明。

　　各位代表，当前我市正处于转型发展、创新创业、爬坡过坎的关键时期，尽管发展中的困难和问题很多，但只要我们坚定信心、奋发有为，就没有迈不过去的坎；只要我们团结一致、凝心聚力，就没有闯不过去的关。让我们在市委的坚强领导下，同心同德、奋力拼搏，全身心投入到第三次创业大潮中，为把我市建成祖国北疆亮丽风景线上的璀璨明珠而努力奋斗！
